# Current Issues, Challenges, and Future Perspectives in Clinical Laboratory Medicine

**DOI:** 10.3390/jcm11030634

**Published:** 2022-01-26

**Authors:** Ferdinando Mannello, Mario Plebani

**Affiliations:** 1 Department of Biomolecular Sciences-DISB, University of Urbino Carlo Bo, 61029 Urbino, Italy; 2 Department of Medicine-DIMED, University of Padua, 35128 Padua, Italy

Laboratory medicine has undergone a profound evolution in organizational, methodological, and cultural terms in recent decades [1]. From the organizational point of view, we are living in the era of consolidation, i.e., the formation of networks of consolidated laboratories with marked automation and integration of the various branches of laboratory medicine [2]. From a methodological point of view, the advent of high-throughput technologies has allowed us to launch a systematic approach to studying nucleic acids, proteins, and intermediate metabolites, all aspects that have considerably reduced the barriers between various branches of biology, to convey all of the information obtained (i.e., the so-called Big Data) into a new perspective of life science related to the biology of systems [3].

In this context, the “Omics” revolution, including mainly genomics, proteomics, degradomics, and metabolomics, has developed into the current major drivers of the bench-to-bedside passage of Omics without limiting the numerous other Omics that opened new and interesting perspectives in laboratory medicine and translational medicine (such as transcrittomics, mirnomics, epigenomics, interactomics, etc.) [4].

The enormous amount of data (“Big Data”) already obtained and still obtainable with Omics analyses have highlighted the professional nature of bioinformatics, opening new perspectives in studying crucial aspects of clinical laboratory medicine: the association–causality relationship; the management of results; the harmonization of data from different technological platforms; and ethical, legal, and privacy issues. Thanks to the use of Omics, clinical laboratory medicine will play a key role in significantly and substantially implementing precision medicine, in preventive screenings, in Omics diagnostics, in personalized drug treatments, and in clinical outcome monitoring.

Through the different Omics branches of clinical laboratory medicine, it will therefore be possible to develop innovative methods in diagnostics, the identification of new diagnostic and/or prognostic biomarkers, the development of innovative target-specific therapies, the design and construction of controlled clinical trials on new drugs, the drafting of new guidelines (such as those already carried out in the field of cardiovascular, hematological, and oncological diseases), as well as both the diagnostics and therapeutic treatments of several human pathologies. All of these crucial aspects are increasingly linked to the concept of well-being, including the application of Omics in laboratory medicine studies on the effects of physical exercise.

Clinical laboratory medicine will therefore change its paradigm, moving away from simple services for clinics and physicians and becoming an even more efficient reference for the diagnosis and treatment of patients [5].

The new diagnostic and therapeutic pathways offered by clinical laboratory medicine are mainly based on the three crucial aspects of appropriateness: prescription, analytics, and diagnostics. Prescriptive appropriateness provides physicians with a constant comparison with other laboratory colleagues to build the right diagnostic protocols. These joint protocols pave the way for feedback, with the best opportunities for updated investigations that the laboratory can offer to the patient, using the best choice of tests (diagnostic settings for personalized and precision medicine) [4].

Analytical appropriateness represents a fundamental part of the status of clinical laboratory medicine specialists as the search for even better technologies and possible new diagnostic tests (based on scientific evidence and surpassing the obsolete ones); this path allows us the best use of financial resources avoiding wasted costs and technologies, focusing efforts according to efficiency, expertise, and targeted epidemiological characteristics of the patient [5].

It is in this perspective that the diagnostic appropriateness must not only have an economic value for cost limitation but also an ethical value for the best diagnostic–therapeutic path of the patient.

Finally, diagnostic appropriateness is mainly aimed at improving clinical outcomes. Only in the face of a constant comparison between the treating physicians and the specialists of laboratory medicine will it be possible to understand the mantra “do the right test to the right patient at the right time and with the right specialist”: in this way, the expected results of diagnostic–therapeutic biomarkers will be obtained with the new Omic approaches of laboratory medicine [6].

If a health system with the patient at the center is oriented towards personalized and/or precision medicine, one cannot ignore appropriateness from a holistic perspective and therefore the indispensable involvement of specialists in laboratory medicine disciplines [5].

In this context, even the pharmaceutical and diagnostic industries can offer a substantial contribution to recovering efficiency and can ensure suitable results, supporting a paradigmatic shift. In fact, diagnostic appropriateness primarily means being able to choose technological innovation (both related to and not related to automation) and laboratory tests with new generation biomarkers on evidence-based medicine.

Importantly, diagnostic appropriateness is born from the definition of guidelines that identify the appropriate tests for a therapy of that type of patient with a specific pathology (and not misunderstanding appropriateness as a mere reduction in financial costs and medical prescriptions by limiting the choices of both clinicians and laboratory specialists in managing the patient’s health) [6].

The best indicator of appropriateness is the state of health that is reachable by the patient through innovation and technologies, simply evaluated in a timely and efficient manner according to a structured path of health technology assessment.

The specific application of guidelines, primary and secondary prevention interventions, initiative medicine and early diagnosis in subjects at risk, and management of chronic (pluri-pathological) patients are just some examples of appropriateness, i.e., appropriate application of health care for both healthy subjects and patients, and a correct interpretation of the holistic concept of health.

If the future of clinical laboratory medicine is precision and personalized medicine, we cannot ignore the appropriateness of diagnostic test requests and therefore the involvement of specialists in the disciplines of laboratory medicine areas in defining optimal diagnostic–therapeutic pathways for patient’s health [7].

On these bases, with great pleasure, we invite specialists from the various branches of laboratory medicine to participate in the submission of scientific work in the fields of clinical chemistry and translational medicine and from the full spectrum of clinical biochemistry and clinical laboratory medicine, promoting excellence in laboratory sciences and closely related fields and sub-specialties. We welcome contributions that will have an impact on the understanding of health and disease and on the progress in basic and applied research in clinical laboratory medicine, taking into consideration papers about all aspects of clinical chemistry and laboratory medicine, with a focus on analytical, preclinical, and clinical investigations of laboratory tests used for diagnosis, prognosis, treatment and therapy, and monitoring of disease in humans.

Welcome to the new “Clinical Laboratory Medicine” section (https://www.mdpi.com/journal/jcm/sectioneditors/clinical_laboratory_medicine accessed on 26 January 2022).
